# Pan-cancer analysis of SERPINE family genes as biomarkers of cancer prognosis and response to therapy

**DOI:** 10.3389/fmolb.2023.1277508

**Published:** 2024-01-11

**Authors:** Yating Liu, Xinyu Li, Shanshan Chen, Changyu Zhu, Yijun Shi, Shoutao Dang, Weitao Zhang, Wei Li

**Affiliations:** Department of Cancer Center, Beijing Tongren Hospital, Capital Medical University, Beijing, China

**Keywords:** SERPINE family genes, pan-cancer, prognosis, tumor microenvironment, immune infiltration, bioinformatics

## Abstract

**Background:** Serine protease inhibitor E (SERPINE) family genes participate in the tumor growth, cancer cell survival and metastasis. However, the SERPINE family members role in the prognosis and their clinical therapeutic potentials in various human cancer types have not been elaborately explored.

**Methods:** We preliminarily analyzed expression levels and prognostic values of SERPINE family genes, and investigated the correlation between SERPINEs expression and tumor microenvironment (TME), Stemness score, clinical characteristic, immune infiltration, tumor mutational burden (TMB), immune subtype, and drug sensitivity in pan-cancer, which based on updated public databases and integrated some bioinformatics analysis methods. In addition, we conducted the enrichment analysis of SERPINEs from DAVID and KOBAS databases.

**Results:** SERPINE1, SERPINE2, and SERPINE3 expression were upregulated in nine cancers, twelve cancers, and six cancers, respectively. The expression of SERPINE family genes was associated with the prognosis in several cancers from The Cancer Genome Atlas (TCGA). Furthermore, SERPINE family genes expression also had a significant relation to stromal and immune scores, and RNA stemness score and DNA stemness score in pan-cancer. SERPINE1 and SERPINE2 expression significantly increased in tumor advanced stage in colon adenocarcinoma (COAD). Results showed that SERPINE1 and SERPINE2 expression were negatively related with B cells and Monocytes, respectively. SERPINE2 expression had a significantly positive relation with B cells and Macrophages. In terms of TMB, SERPINE1, SERPINE2, and SERPINE3 were found to associated with TMB in seven cancers, fourteen cancers, and four cancers, respectively. Moreover, all SERPINE gene family members were significantly correlated with immune subtypes. SERPINE1 expression had a significantly positive or negative correlation with drug sensitivity.

**Conclusion:** The study indicated the great potential of SERPINE family genes as biomarkers for prognosis and provided valuable strategies for further investigation of SERPINE family genes as potential targets in cancer.

## 1 Introduction

Cancer is a major public health problem worldwide. According to the Global Cancer Statistics 2020, there will be an estimated 19.3 million new cancer cases and almost 10.0 million cancer deaths occurred in 2020 (Sung et al., 2021). The burden of cancer incidence and mortality is rapidly growing worldwide. Cancer is a genetic disease resulting from the accumulation of mutations in genes that regulate cell division, survival, invasion or other hallmarks of the transformed phenotype (Chakravarty and Solit, 2021). Genomic analyses in cancer have been enormously impactful, leading to the identification of driver mutations and development of targeted therapies (Mani et al., 2022). However, despite the advances in treatment options based on targeted therapies for specific types of cancer, only a few cancers have targetable molecules. In addition, tumor resistance to molecularly targeted therapies limits the effectiveness of current cancer therapies (Holohan et al., 2013). Therefore, it is essential to explore more molecular targets to optimize combination therapy and increase these success rates.

Hypoxia is one of the crucial features of the tumor microenvironment (TME), which can directly result to the malignant characteristics, including tumor proliferation, migration and invasion, and lead to poor prognosis (Wilson and Hay, 2011; Gilkes et al., 2014; Muz et al., 2015; Shida et al., 2016). Hypoxia-inducible factor-1(HIF-1) is a key transcription factor in cellular adaptation to hypoxia (Harris, 2002; Semenza, 2014). HIF-1 consists of an oxygen-sensitive α-subunit (HIF-1α) and a constitutive β-subunit (HIF-1β). HIF-1α are quickly activated in hypoxia and are responsible for transcription and expression of plasminogen activator inhibitor-1 (PAI-1) (Liao et al., 2007; Samoylenko et al., 2010). The overexpression of HIF-1α is associated with an aggressive phenotype and poor prognosis in multiple tumors (Semenza, 2002; Ferrara et al., 2003).

Serine protease inhibitor E (SERPINE), as a member of the serpin superfamily, has the structure of three β-sheets (A, B, and C) and nine α-helices (hA through hI) (Irving et al., 2000; Gettins and Olson, 2016). The SERPINE family has three members: SERPINE1 (also known as PAI-1), SERPINE2 (also known as protease nexin-CC1, PN-1), and SERPINE3. Recently, Zhang et al. demonstrated that hypoxia-induced reactive oxygen species (ROS) reinforce the hypoxic adaptation of glioblastoma by driving the HIF-1α-SERPINE1 signaling pathway (Zhang et al., 2023). Pei et al. developed a hypoxia risk score model based on SERPINE1 revealing the correlation between hypoxia and tumor immune microenvironment (Pei et al., 2021). In addition, numerous studies published in the past few decades reported that SERPINE1 is one of the most reliable biomarkers and prognostic markers in many cancer types, including breast cancer (Harbeck et al., 2004; Palmirotta et al., 2009; Duffy et al., 2014; Duffy et al., 2016; Jevrić et al., 2019), ovarian cancer (Mashiko et al., 2015; Nakatsuka et al., 2017), renal cell carcinoma (Ahluwalia et al., 2021), head and neck squamous cell carcinoma (Pavón et al., 2015; Guo et al., 2022; Zhou et al., 2022; Li et al., 2023), bladder cancer (Chan et al., 2017), colorectal cancer (Sakakibara et al., 2005), gastric cancer (Khan et al., 2022; Xu et al., 2022) and non-small cell lung cancers (Sotiropoulos et al., 2019). Previous studies also demonstrated that SERPINE1 can promote cancer progression and metastasis as it is promoting the tumor migration, invasion, and angiogenesis (Dellas and Loskutoff, 2005; Li et al., 2018; Kubala and DeClerck, 2019). SERPINE1 is generally classified as a hub or core gene in a wide spectrum of cancer types. For many of the poor outcome cancers, identification of SERPINE1 in the complement of hub or signature genes is a strong indicator of reduced patient survival (Czekay et al., 2022). Several studies have shown that SERPINE2 expression is associated with tumorigenesis and tumor cell invasion (Wagenblast et al., 2015; Monard, 2017). SERPINE2 is involved in the progression of some cancers, including lung cancer (Dokuni et al., 2020; Zhang et al., 2022a), renal cell carcinoma (Chen et al., 2023), esophageal squamous cell carcinoma (Zhang et al., 2020), colorectal cancer (Bergeron et al., 2010), pancreatic tumors (Buchholz et al., 2003), melanoma (Perego et al., 2018; Kang et al., 2022), and breast cancer (Wagenblast et al., 2015; Smirnova et al., 2016). For example, SERPINE2 overexpression promoted the metastasis of breast cancer by remodeling the tumor matrix (Zhou et al., 2023). The research of SERPINE3 in human cancers is largely unexplored. Therefore, SERPINE family genes are significant regulators in various human cancers, and explore the full picture of SERPINE family in pan-cancer is valuable and necessary for a better understanding of their roles in cancer development and their clinical therapeutic potentials. Additionally, the importance of hypoxia in promoting tumor immune escape and immunosuppression has received increasing attentions (Pei et al., 2021). It is significant to explore the potential connections between hypoxia and tumor immune microenvironment.

In this research, we primarily investigated the basal expression levels of SERPINE family genes in human cancer samples and human normal tissues from The Cancer Genome Atlas (TCGA) database. Then, we comprehensively investigated the prognostic value of SERPINEs in pan-cancer via multiple databases, including TCGA and Kaplan-Meier Plotter. Besides, we also explored the association between SERPINE family gene expression and TME, Stemness score, clinical stages, tumor mutational burden (TMB), and immune infiltration cells. We predicted the possible pathways that SERPINEs participates in tumorigenesis by enrichment analysis. Moreover, we analyzed the relation between SERPINE expression and drug sensitivity and immune subtype in human cancers. In general, our study comprehensively evaluated the SERPINEs expression levels and prognostic values in pan-cancer, and provide new insights for SERPINE family genes as potential treatment targets in various cancers.

## 2 Materials and methods

### 2.1 Gene expression analysis

We downloaded the gene expression RNAseq (FPKM format), clinicopathological data, immune subtype, survival data, stemness score (RNA based), and (DNA methylation-based) of 33 cancers from TCGA database using UCSC Xena (https://xena.ucsc.edu/) (Goldman et al., 2020). The mRNA expression profile with normal tissue was extracted from Genotype-Tissue Expression (GTEx) database (https://gtexportal.org/home/datasets) to supply normal tissue RNA-seq transcriptome data lacking in TCGA. The abbreviations and full names of the cancers involved in the study are presented in Table 1. The perl software was used to extract and integrate the SERPINE family genes expression level. The “Wilcox. test” was also employed to assess the differential SERPINE family gene expression in 33 cancer types. “*,” “**,” “***,” indicate *p-*value <0.05, <0.01, <0.001, respectively. The pictures of the analyses results were drawn with the R-package “ggplot.” Besides, a box plot and heatmap were designed using the “ggpubr” and “pheatmap” R-packages, respectively, for illustrating the expression pattern of SERPINE family genes. The R-package “corrplot” was employed for the correlation analysis among SERPINE family genes. In order to investigate differences in SERPINEs protein expressions between normal and tumor tissues, the UALCAN database (http://ualcan.path.uab.edu), which performs protein expression analysis based on the data from the Clinical Proteomic Tumor Analysis Consortium (CPTAC), was employed.

**TABLE 1:** The 33 tumors analyzed in this study.

Full names	Abbreviations
Adrenocortical carcinoma	ACC
Bladder Urothelial Carcinoma	BLCA
Breast invasive carcinoma	BRCA
Cervical squamous cell carcinoma and endocervical adenocarcinoma	CESC
Cholangiocarcinoma	CHOL
Colon adenocarcinoma	COAD
Lymphoid neoplasm diffuse large B-cell lymphoma	DLBC
Esophageal carcinoma	ESCA
Glioblastoma multiforme	GBM
Head and neck squamous cell carcinoma	HNSC
Kidney chromophobe	KICH
Kidney renal clear cell carcinoma	KIRC
Kidney renal papillary cell carcinoma	KIRP
Acute myeloid leukemia	LAML
Brain lower grade glioma	LGG
Liver hepatocellular carcinoma	LIHC
Lung adenocarcinoma	LUAD
Lung squamous cell carcinoma	LUSC
Mesothelioma	MESO
Ovarian serous cystadenocarcinoma	OV
Pancreatic adenocarcinoma	PAAD
Pheochromocytoma and paraganglioma	PCPG
Prostate adenocarcinoma	PRAD
Rectum adenocarcinoma	READ
Sarcoma	SARC
Skin cutaneous melanoma	SKCM
Stomach adenocarcinoma	STAD
Testicular germ cell tumors	TGCT
Thyroid carcinoma	THCA
Thymoma	THYM
Uterine corpus endometrial carcinoma	UCEC
Uterine carcinosarcoma	UCS
Uveal Melanoma	UVM

### 2.2 Survival analysis of expression of SERPINE family genes

Survival data for each sample was acquired from TCGA database, which was used to analyzed the relationship between expression of SERPINE family genes and clinical outcome. A total of four survival prognosis indexes, including Overall Survival (OS), Disease-Specific Survival (DSS), Disease-Free Interval (DFI), and Progression-Free Interval (PFI) were evaluated. The survival analysis (*p* < 0.05) in different cancers was utilized by R-package “survminer” and “survival” and the “high” and “low” subgroup was depended on the cutoff value of SERPINEs expression, and was shown by the Kaplan-Meier plot. Besides, the association between the SERPINE family genes expression and prognosis of pan-cancer was established by a COX analysis. Finally, the forest plot was drawn using “forestplot” and “survival” packages. Furthermore, online database Kaplan-Meier Plotter (https://kmplot.com/analysis/) was employed to verify the relationship between SERPINE family genes expression and survival in pan-cancer to identify its association with clinical outcome (OS). We applied Kaplan-Meier Plotter database [originated from TCGA, gene expression omnibus (GEO) and European Genome-phenome Archive (EGA)] to evaluate the effect of the SERPINE family genes on the survival of patients affected by 21 different tumors.

### 2.3 Tumor immune microenvironment and stemness score analysis

Tumor immune microenvironment consists of tumor cells, tumor stromal cells including stromal fibroblasts, endothelial cells and immune cells like microglia, macrophages and lymphocytes and the non-cellular components of extracellular matrix such as collagen, fibronectin, hyaluronan, laminin, among others (Baghban et al., 2020). The comprehensive analysis of the multiple exchanges between tumor cells and tumor immune microenvironment is necessary to understand the different underlying mechanisms of cancer growth and metastasis (Jahanban-Esfahlan et al., 2018). The ESTIMATE algorithm in R-package “estimate” and “limma” were employed to calculate stromal and immune cell scores in each tumor based on gene expression in different cancers. The Spearman’s method was applied to analyze the correlation between SERPINE family genes expression and RNA stemness score (RNAss), DNA stemness score (DNAss), which were visualized by the “corrplot” package. Moreover, the “reshape2,” “ggpubr,” “ggplot2” and “limma” packages were employed to conduct a correlation analysis of SERPINE family genes expression with the TME and stemness score in selected cancers.

### 2.4 Clinical characteristics analysis

Correlation analysis between clinical characteristics and SERPINE family gene was performed in selected tumors. Firstly, the R-package “ggplot2” was applied to the examination for the relationship between the clinical stages and SERPINE family gene in selected cancers [kidney chromophobe (KICH), head and neck squamous cell carcinoma (HNSC), colon adenocarcinoma (COAD), and breast invasive carcinoma (BRCA)] from TCGA database. In addition, we also downloaded selected cancer types (HNSC and COAD) data and the corresponding clinical datasets (GSE65858 and GSE40967) from the Gene Expression Omnibus (GEO; https://www.ncbi.nlm.nih.gov/geo/) to identify the clinical characteristic analysis. We employed “ggpubr” package to identify the correlation between the clinical stages and SERPINE family genes in selected cancers (HNSC and COAD) from GEO database.

### 2.5 Immune infiltration cells and TMB analysis

In order to explore the relationship between SERPINE family genes expression and tumor infiltration immune cells level in pan-cancer, the R-package “ggplot2,” “ggpubr,” and “ggExtra” were used, and we also applied the tool CIBERSORT to estimate immune infiltration data from the ImmuCellAI database (http://bioinfo.life.hust.edu.cn/ImmuCellAI#!/). In addition, the association between SERPINE family genes expression and TMB were analyzed by using R package “fmsb” and Pearson correlation was calculated, with these findings displayed in the form of radar plots.

### 2.6 Enrichment analysis

We used OPEN TARGET platform (https://www.target-validation.org/) to explore SERPINE family genes related diseases network (Carvalho-Silva et al., 2019). In addition, the DAVID database and KOBAS database were applied to Gene Ontology (GO) functional analysis and Kyoto Encyclopedia of Genes and Genomes (KEGG) pathway analysis. Finally, the Chinese bioinformatics website (http://www.bioinformatics.com.cn/) was employed to do the analysis including GO functional analysis and KEGG pathway analysis.

Furthermore, the proteins interacting with SERPINE1 protein were conducted using the STRING (https://string-db.org/) open database. Differential genes were obtained by differential analysis of selected cancers (HNSC, COAD, and BRCA; datasets, GSE65858, GSE40967, and GSE42568, respectively) from GEO database. Then, these differential genes were used to construct protein–protein interaction (PPI) network.

### 2.7 Drug sensitivity and immune subtype analysis

We downloaded drug sensitivity processed data from CellMiner™ database (https://discover.nci.nih.gov/cellminer/home.do). The R-package “impute,” “limma,” “ggplot2,” and “ggpubr” were applied to the data process and result visualization. Besides, the R-package “limma,” “ggplot2,” and “reshape2” were used for the immune subtype correlation analysis of SERPINE family genes.

## 3 Results

### 3.1 The expression levels of SERPINE family genes in pan-cancer

**FIGURE 1:**
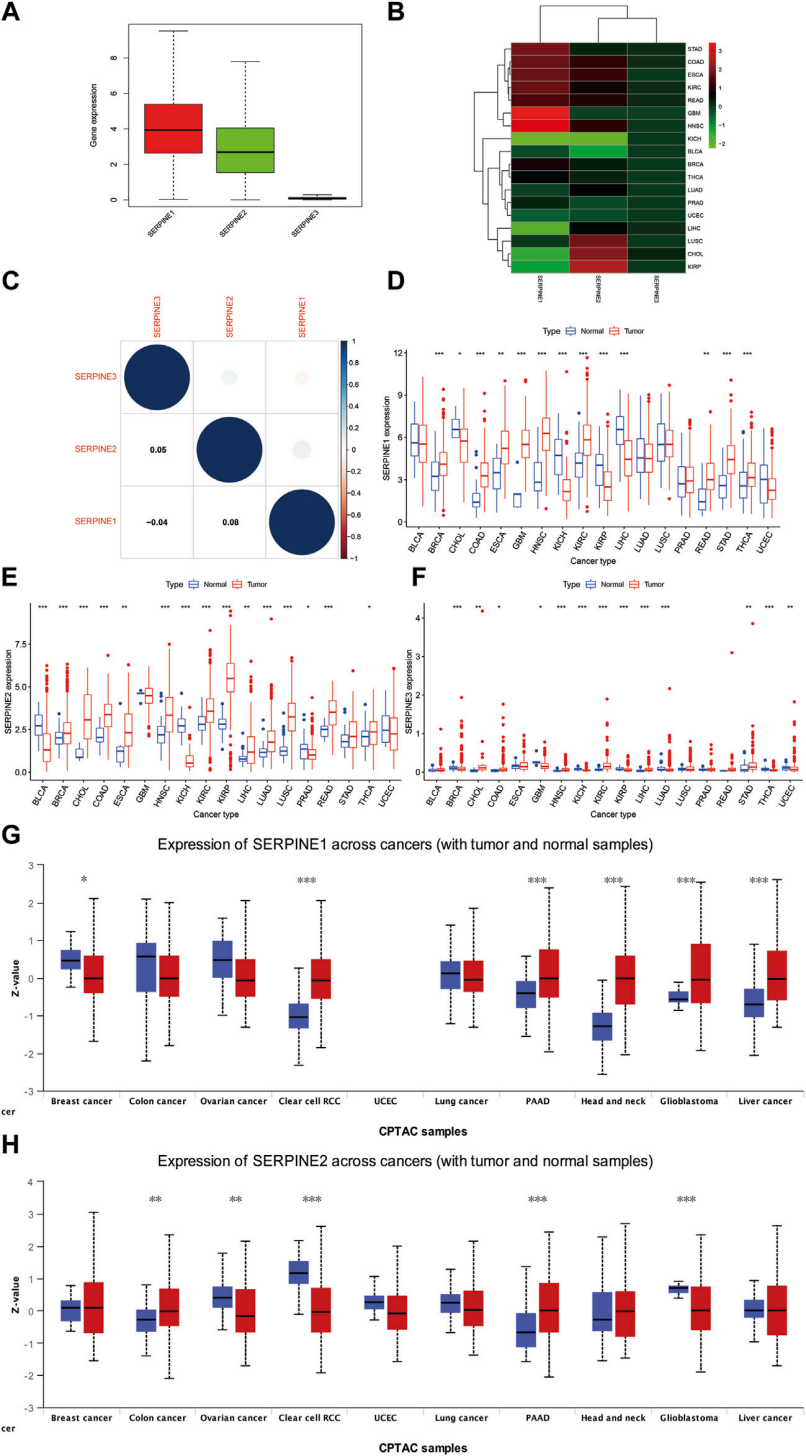
Differences in RNA and protein expression levels of SERPINE family genes in different tumors. **(A)** Boxplot illustrating the distribution of SERPINE family genes expression in various cancers. **(B)** Heatmap showing the difference of SERPINE family genes expression levels in different cancer types from TCGA database. The red and green indicate the high or low expression, respectively. **(C)** The correlation between SERPINE family genes. **(D–F)** The expression level of SERPINE family genes in normal and tumor tissues in human pan-cancer. **(G–H)** The different protein expression level in matched tumor tissues in the UALCAN database. The blue boxplots indicate the normal tissues and the red boxplots indicate the cancer tissues. Z-values represent standard deviations from the median across samples for the cancer types. (*p* < 0.05 was considered significant, *p < 0.05, **p < 0.01, and ***p < 0.001).

Our results indicated that SERPINE1 was highly expressed, SERPINE2 was moderately expressed, and SERPINE3 was lowly expressed in pan-cancer (Figure 1A). SERPINE family genes expression and correlation in various cancer types were further analyzed by TCGA database. The results shown that SERPINE1 was the highest expression in glioblastoma multiforme (GBM), SERPINE2 was the highest expression in kidney renal papillary cell carcinoma (KIRP), and SERPINE3 was the highest expression in cholangiocarcinoma (CHOL) (Figure 1B). In addition, SERPINE1 and SERPINE2 were the two genes with the most significant positive correlation, whereas SERPINE1 and SERPINE3 were the two genes with negative correlation (Figure 1C).

We then used R software to analyze RNA sequencing data from TCGA database to estimate SERPINE family genes differential expression in pan-cancer. Our results signified that SERPINE1 was more expressed in BRCA, COAD, GBM, HNSC, kidney renal clear cell carcinoma (KIRC), stomach adenocarcinoma (STAD), thyroid carcinoma (THCA), esophageal carcinoma (ESCA), rectum adenocarcinoma (READ). In contrast, a lower SERPINE1 expression was found in KICH, KIRP, liver hepatocellular carcinoma (LIHC), CHOL (Figure 1D). SERPINE2 was more expressed in several cancers, including BRCA, CHOL, COAD, HNSC, KIRC, KIRP, lung adenocarcinoma (LUAD), Lung squamous cell carcinoma (LUSC), READ, ESCA, LIHC, THCA. Meanwhile, SERPINE2 was lower expressed in Bladder Urothelial Carcinoma (BLCA), KICH, Prostate adenocarcinoma (PRAD) (Figure 1E). The higher expression of SERPINE3 was discovered in HNSC, KIRC, LIHC, CHOL, STAD, COAD. However, the lower expression was in BRCA, KICH, KIRP, LUAD, THCA, uterine corpus endometrial carcinoma (UCEC), GBM (Figure 1F). Due to the normal tissue controls were very limited in some cancer types, we then integrated TCGA and GTEx databases to investigate the differential expression of SERPINEs (Supplementary Figure S1). We found SERPINE1 was highly expressed in cancer tissues in BRCA, GBM, brain lower grade glioma (LGG), pancreatic adenocarcinoma (PAAD), ESCA, STAD, COAD, READ, testicular germ cell tumors (TGCT), Thymoma (THYM), Lymphoid neoplasm diffuse large B-cell lymphoma (DLBC), KIRC, and HNSC. SERPINE1 was lower expressed in ovarian serous cystadenocarcinoma (OV), UCEC, adrenocortical carcinoma (ACC), THCA, LUSC, LUAD, SKCM, PRAD, LIHC, KICH, KIRP, acute myeloid leukemia (LAML), and CHOL. In addition, SERPINE2 was higher expressed in UCEC, Uterine carcinosarcoma (UCS), BRCA, LGG, GBM, ACC, LUAD, LUSC, PAAD, STAD, SKCM, COAD, READ, THYM, DLBC, LIHC, KIRP, KIRC, LAML, CHOL, HNSC, and Pheochromocytoma and paraganglioma (PCPG). The lower expression of SERPINE2 was found in OV, THCA, PRAD, KICH, BLCA, and CESC. SERPINE3 was highly expressed in THYM, KIRC, LAML, CHOL, as well as HNSC, and lower expressed in OV, UCS, UCEC, BRCA, LGG, GBM, THCA, LUAD, LUSC, PAAD, SKCM, READ, COAD, PRAD, TGCT, LIHC, KICH and KIRP.

To assess the differences in SERPINEs at the translational level, the CPTAC database was used to compare differences in SERPINEs protein levels between normal and tumor groups on the UALCAN website. It was evident that the level of SERPINE1 protein was higher in HNSC, KIRC, LIHC, PAAD and GBM. Compared to HNSC, KIRC and GBM, variations in SERPINE1 protein levels in BRCA, LIHC, and PAAD were not in tandem with SERPINE1 RNA expressions (Figure 1G). The results demonstrated that SERPINE2 protein expression was significantly upregulated in COAD and PAAD tumor tissues in a comparison with a normal one (Figure 1H, *p* < 0.05).

### 3.2 Prognostic value of SERPINE family genes in pan-cancer

**FIGURE 2:**
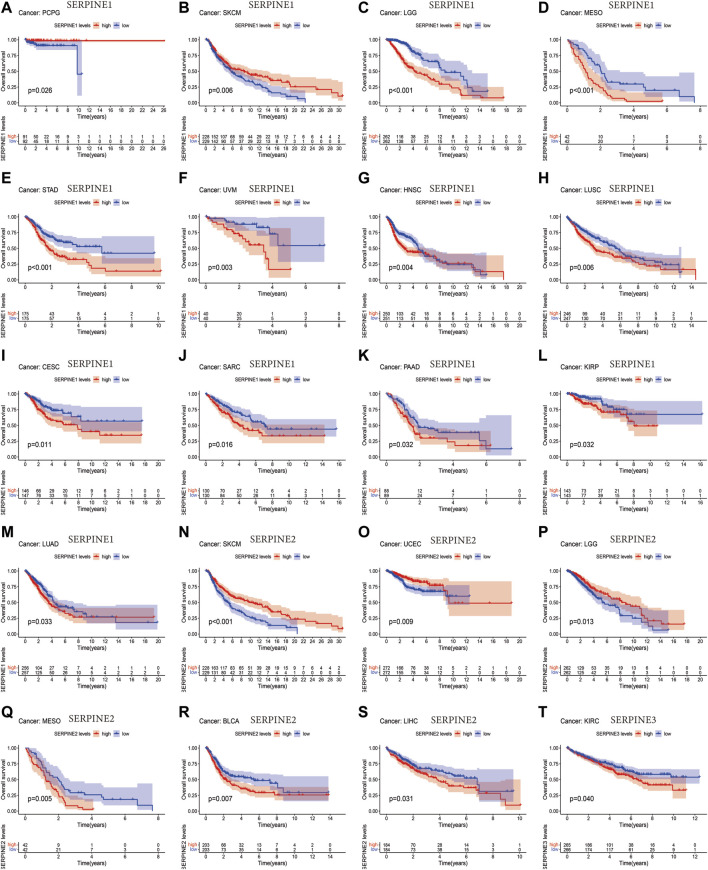
Survival analysis of SERPINE family genes across multiple cancer types. The red line in the photos indicates high expression and the blue line in the photos indicates low expression. *p*-value less than 0.05 is considered as difference.

Next, we employed different databases to verify the prognostic value of the SERPINE family genes in pan-cancer. Kaplan-Meier survival curves demonstrated that SERPINE family genes expression was related to the prognosis in several cancer types (Figure 2). SERPINE1 was a protective factor in UCEC. While, SERPINE1 had a detrimental role in LGG, mesothelioma (MESO), STAD, uveal melanoma (UVM), HNSC, LUSC, cervical squamous cell carcinoma and endocervical adenocarcinoma (CESC), sarcoma (SARC), PAAD, KIRP, LUAD. SERPINE2 played a protective role in SKCM, UCEC, LGG. At the same time, SERPINE2 was a high-risk gene in MESO, BLCA, LIHC. Besides, SERPINE3 was a dangerous factor in KIRC.

**FIGURE 3:**
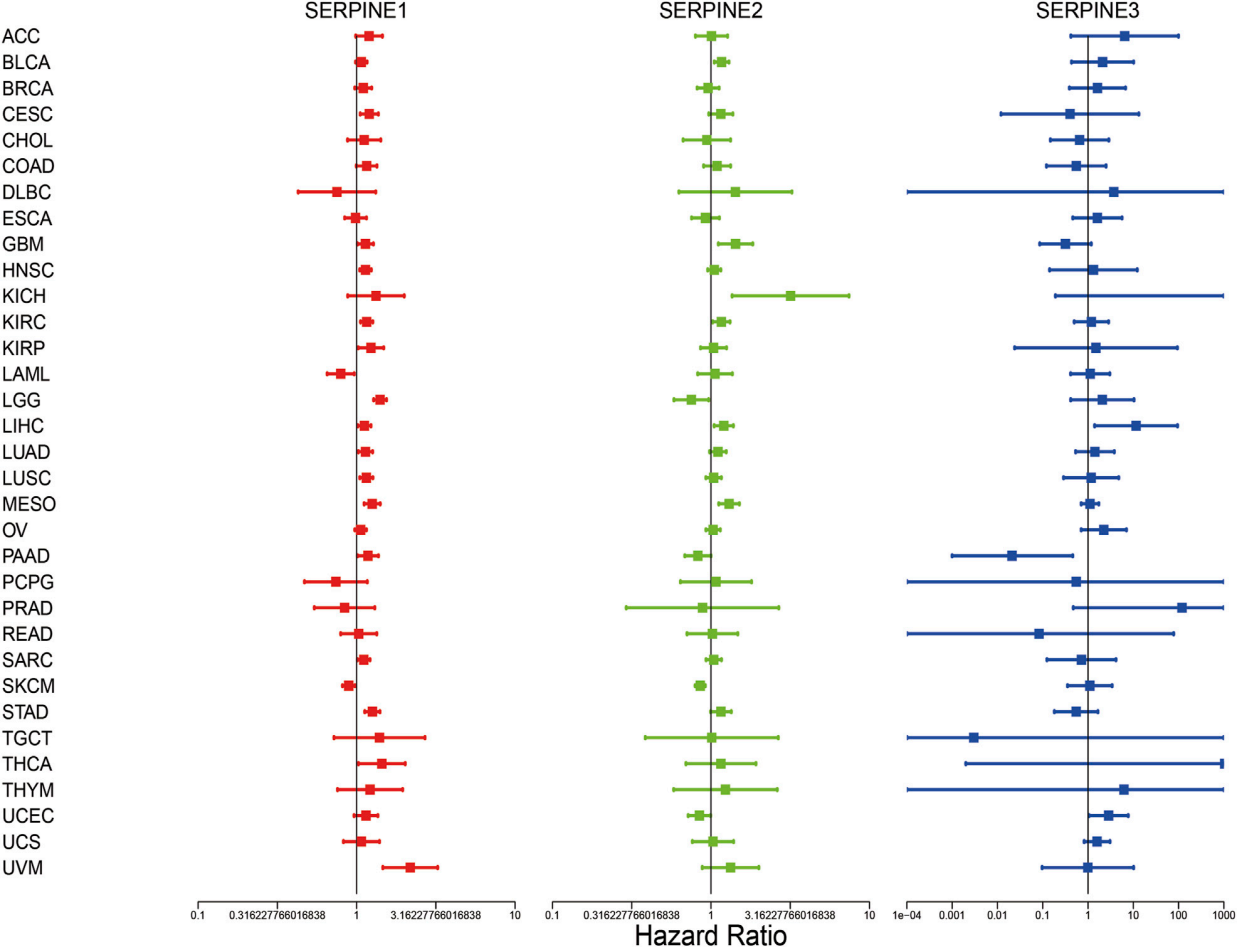
Correlation analysis of SERPINE family genes expression with survival by the COX method in different types of cancers. Hazard ratio <1 represent low risk and hazard ratio >1 represent high risk. (*p* < 0.05 was considered significant).

Then, we investigated the prognosis risk of SERPINE family genes in pan-cancer via COX analysis (Figure 3; Table 2). SERPINE1 was a low-risk factor in SKCM, LAML. However, SERPINE1 was a high-risk factor in LGG, STAD, UVM, MESO, KIRC, HNSC, LUSC, CESC, LUAD, LIHC, SARC, KIRP, GBM, PAAD, and thyroid carcinoma (THCA). SERPINE2 had a protective effect in SKCM, LGG, UCEC. In contrast, SERPINE2 acted as a detrimental factor in MESO, BLCA, GBM, KICH, LIHC, KIRC. SERPINE 3 played a protective role in PAAD. For another, SERPINE3 functioned as a high-risk gene in LIHC, UCEC. The results demonstrated that SERPINE family genes may act as different roles in prognosis value in different cancers.

**TABLE 2:** SERPINE family genes were associated with the prognosis risks of different cancers by COX analysis.

Gene	Cancer	HR	HR.95L	HR.95H	*p*-value
SERPINE1	SKCM	0.892	0.815	0.976	0.0130
	LAML	0.794	0.652	0.966	0.0215
	LGG	1.406	1.281	1.544	7.08E-13
	STAD	1.258	1.126	1.404	4.50E-05
	UVM	2.181	1.464	3.248	0.0001
	MESO	1.254	1.116	1.409	0.0001
	KIRC	1.158	1.059	1.265	0.0012
	HNSC	1.138	1.047	1.238	0.0025
	LUSC	1.152	1.048	1.266	0.0035
	CESC	1.202	1.054	1.372	0.0063
	LUAD	1.138	1.026	1.262	0.0147
	LIHC	1.120	1.018	1.232	0.0196
	SARC	1.110	1.014	1.215	0.0243
	KIRP	1.232	1.023	1.482	0.0274
	GBM	1.137	1.014	1.276	0.0280
	PAAD	1.180	1.014	1.373	0.0322
	THCA	1.443	1.027	2.027	0.0347
SERPINE2	SKCM	0.856	0.795	0.921	3.42E-05
	LGG	0.752	0.584	0.967	0.0266
	UCEC	0.845	0.718	0.995	0.0434
	MESO	1.301	1.120	1.511	0.0006
	BLCA	1.167	1.049	1.298	0.0045
	GBM	1.429	1.113	1.834	0.0051
	KICH	3.178	1.360	7.427	0.0076
	LIHC	1.204	1.047	1.384	0.0093
	KIRC	1.163	1.025	1.318	0.0186
SERPINE3	PAAD	0.021	0.001	0.459	0.0141
	LIHC	11.525	1.399	94.913	0.0231
	UCEC	2.853	1.053	7.731	0.0393

HR, hazard ratio.

**FIGURE 4:**
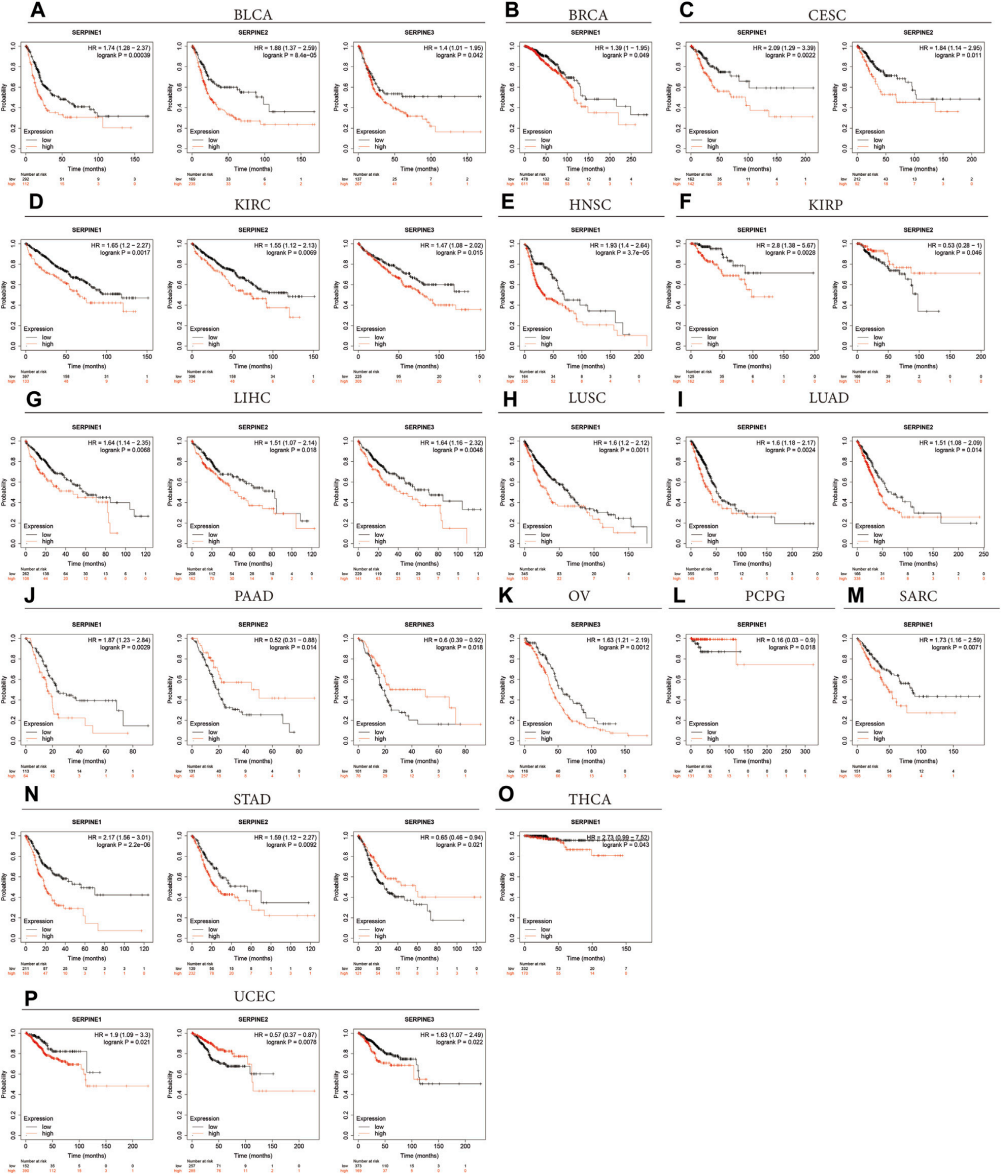
Overall survival curves comparing the high and low expression of SERPINE family genes in various cancers in Kaplan-Meier Plotter database. (*p* < 0.05 was considered significant).

Next, the Kaplan-Meier plotter was employed to assess SERPINE family gene-related survival (OS) based on TCGA, GEO and EGA databases. As shown in Figure 4, all SERPINE gene family members had detrimental effect on BLCA (Figure 4A). The similar results can also be found in KIRC, and LIHC (Figures 4D,G). In addition, SERPINE1 played a detrimental prognostic role in BRCA, HNSC, LUSC, SARC, and THCA (Figures 4B,E,H, M, O). However, SERPINE1 had a protective prognostic role in PCPG (Figure 4L). SERPINE1 and SERPINE2 both had detrimental roles in CESC, and LUAD (Figures 4C,I). For KIRP, SERPINE1 had a detrimental effect, and SERPINE2 acted as a low-risk factor (Figure 4F). SERPINE1 played a detrimental prognostic role in PAAD, and SERPINE2 and SERPINE3 had protective effects on PAAD (Figure 4J). SERPINE3 was a high-risk factor in OV (Figure 4K). Besides, SERPINE1 and SERPINE2 acted detrimental roles in STAD, and SERPINE3 was a protective factor in STAD (Figure 4N).

Some contradictory data related to SERPINE family genes expression was found in some cancers from the above results from different databases (Table 3). The hypothetical mechanisms with different biological characteristics and distinct data collection methods were the cause of these contradictory results.

**TABLE 3:** The correlation between SERPINE family genes high expression and pan-cancer in different database. The clinical outcome is OS.

Gene	Effect	TCGA (Kaplan-Meier)	TCGA (COX)	Kaplan-meier plotter
SERPINE1	Protective	PCPG, SKCM	SKCM, LAML	PCPG
	Detrimental	LGG, MESO, STAD, UVM, HNSC, LUSC, CESC, SARC, PAAD, KIRP, LUAD	LGG, STAD, UVM, MESO, KIRC, HNSC, LUSC, CESC, LUAD, LIHC, SARC, KIRP, GBM, PAAD, THCA	BLCA, BRCA, CESC, KIRC, HNSC, KIRP, LIHC, LUSC, LUAD, PAAD, SARC, STAD, THCA, UCEC
SERPINE2	Protective	SKCM, UCEC, LGG	SKCM, LGG, UCEC	KIRP, PAAD, UCEC
	Detrimental	MESO, BLCA, LIHC	MESO, BLCA, GBM, KICH, LIHC, KIRC	BLCA, CESC, KIRC, LIHC, LUAD, STAD
SERPINE3	Protective	NA	PAAD	PAAD, OV, STAD
	Detrimental	KIRC	LIHC, UCEC	BLCA, KIRC, LIHC, UCEC

OS: overall survival; TCGA: the cancer genome atlas; NA: not available.

### 3.3 Relationship between SERPINE family genes expression and TME, stemness score in pan-cancer

**FIGURE 5:**
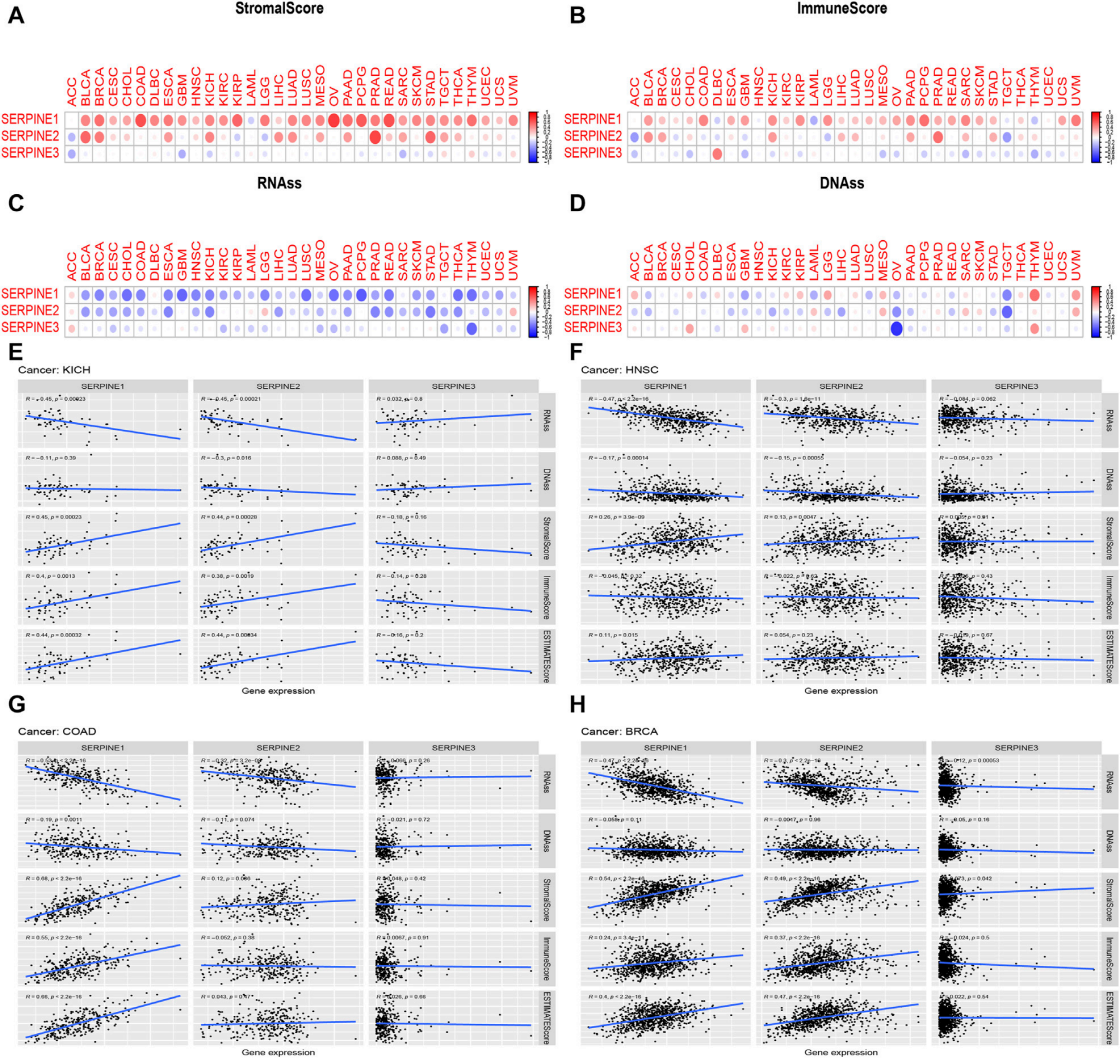
Correlation of SERPINE family genes expression with tumor microenvironment, Stemness score. The SERPINE family genes associated Stromal score **(A)** Immune score; **(B)** RNAss; **(C)** and DNAss **(D)** are illustrated. Red dots indicate a positive correlation, and blue dots indicate a negative correlation. **(E–H)** Correlation analysis of SERPINEs expression with Stemness score, TME in four cancer types. R means correlation value, positive number means positive correlation, negative number means negative correlation. (*p* < 0.05 was considered significant).

We further explored the association between SERPINE family genes expression and TME in pan-cancer. As shown in Figure 5, SERPINE family genes expression had a significantly positive or negative correlation with stromal and immune scores in pan-cancer (Figures 5A, B). Specifically, we found that SERPINE1 and SERPINE2 were significantly positively associated with stromal score and immune score in most cancers. On the other hand, SERPINE3 had a negative relationship in stromal and immune scores in most cancers. Furthermore, most of SERPINE family genes expression was significantly negatively related to RNAss and DNAss (Figures 5C, D). Our results indicated that SERPINE family genes different in their capacity to regulate the immune microenvironment in different tumors.

### 3.4 Association between SERPINE family genes expression and TME, stemness score in selected cancers

By gene expression analysis, we found that SERPINE family genes were all lower expressed in KICH and higher expressed in HNSC, and *p* < 0.001. Besides, SERPINE1 and SERPINE2 were all higher expressed in COAD and BRCA with *p* < 0.001. Thus, we analyzed the relation between SERPINE family genes expression and TME, Stemness score in these four cancer types from TCGA database. As was shown in Figure 5, SERPINE1 and SERPINE2 were negatively associated with RNAss in KICH. Besides, SERPINE2 was negatively related to DNAss in KICH. The expression of SERPINE1 and SERPINE2 were positively related to stromal score in KICH. Similarly, SERPINE1 and SERPINE2 were positively associated with the immune score, and estimate score in KICH (Figure 5E). In addition, the expression of SERPINE1 and SERPINE2 were negatively correlated with RNAss and DNAss in HNSC. However, SERPINE1 and SERPINE2 were positively associated with the stromal score in HNSC. SERPINE1 was positively related to estimate score in HNSC (Figure 5F). The expression of SERPINE1 and SERPINE2 were negatively related with RNAss in COAD. Besides, SERPINE1 had a significantly negative relation with DNAss in COAD. Furthermore, SERPINE1 was positively associated with stromal score, immune score, and estimate score in COAD (Figure 5G). In BRCA, SERPINE1, SERPINE2 and SERPINE3 were negatively related to RNAss. Moreover, SERPINE1, SERPINE2 and SERPINE3 were positively associated with stromal score. The expression of SERPINE1 and SERPINE2 were positively related with the immune score and estimate score in BRCA (Figure 5H).

### 3.5 Correlation between clinical characteristics and SERPINE family genes expression in selected cancers

**FIGURE 6:**
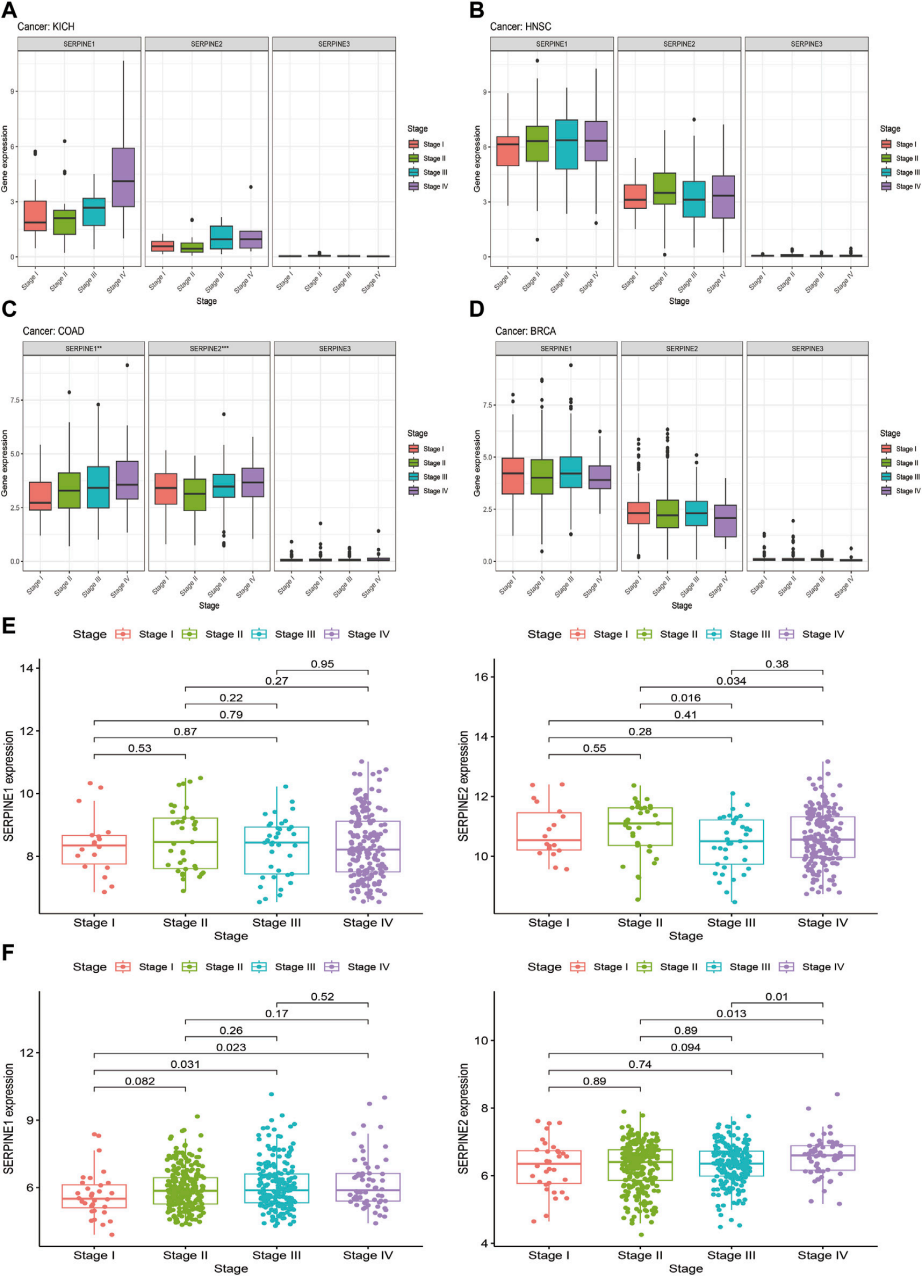
Correlation between SERPINE family genes expression and tumor stages. **(A–D)** Association of SERPINE family genes expression with the clinical stages in selected cancers from TCGA database. **(E,F)** Association of SERPINE family genes expression with the clinical stages in selected cancers from GEO database. (*p* < 0.05 was considered significant, *p < 0.05, **p < 0.01, and ***p < 0.001).

Based on gene expression analysis from TCGA database, four cancer types (KICH, HNSC, COAD, and BRCA) were selected for SERPINE family genes expression levels and tumor stage correlation analysis (Figure 6). We discovered that the expression of SERPINE1 and SERPINE2 were significantly related to tumor stage in COAD. SERPINE1 and SERPINE2 expression significantly increased in tumor advanced stage in COAD. In addition, the GEO database was also employed to verify the association between SERPINE family genes expression and clinical stage in four cancers. Unfortunately, correlation analysis of SERPINE family genes expression and tumor stage could not be performed because the clinical datasets of KICH and BRCA did not meet our requirements. Thus, HNSC and COAD from GEO database were analyzed. Similarity, the expression of SERPINE1 and SERPINE2 increased in advanced stage of COAD.

### 3.6 Correlation between SERPINE family genes expression and immune infiltration and TMB

**FIGURE 7:**
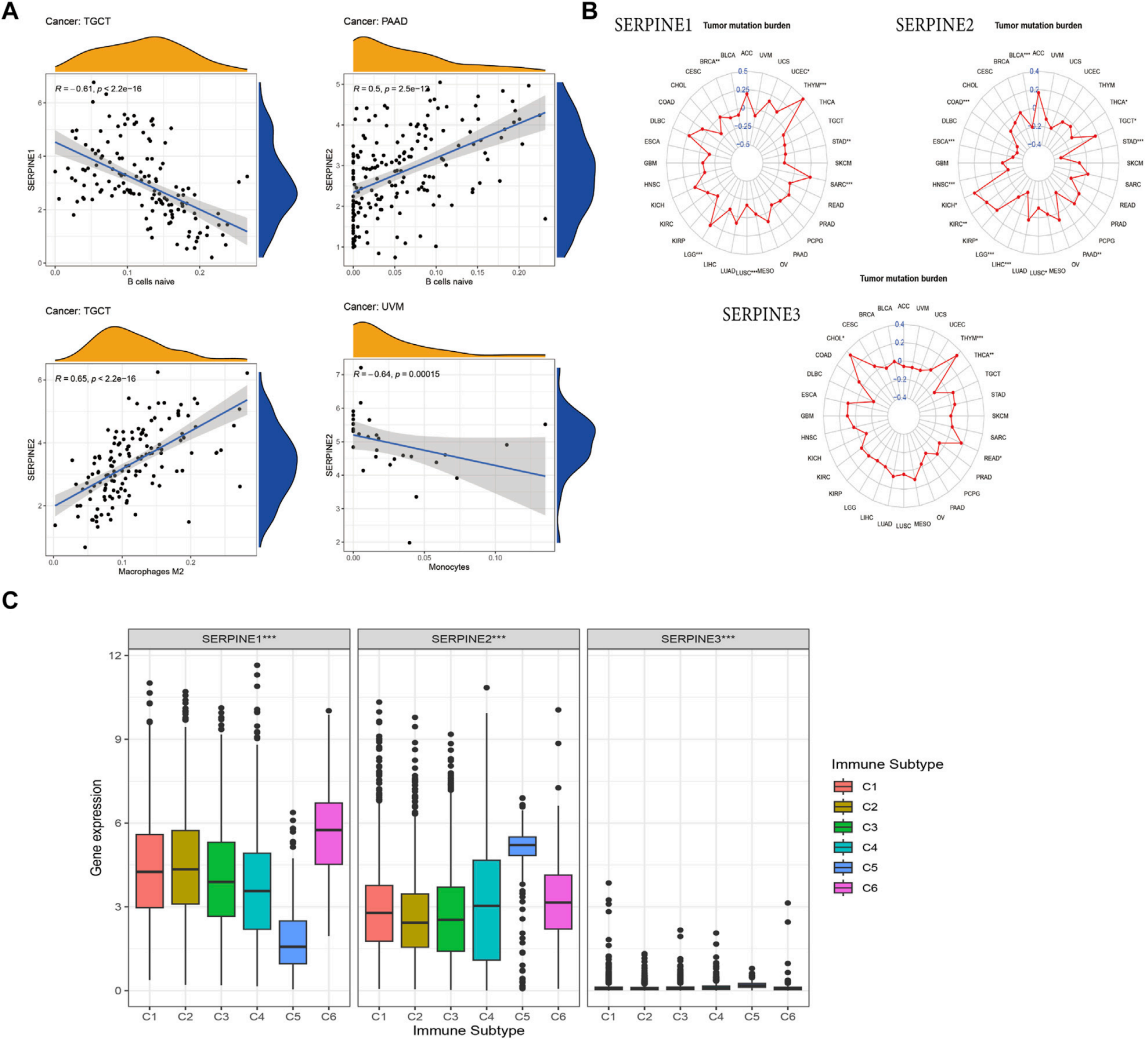
Association of SERPINE family genes expression with immune infiltration, TMB and immune subtype in different cancer types. R > 0.5, *p* < 0.05. **(A)** The correlation between SERPINEs and immune infiltration. **(B)** The correlation between SERPINEs and TMB. **(C)** SERPINE family genes expression levels of different immune subtype in pan-cancer. *X*-axis represents immune subtype, *Y*-axis represents gene expression. C1, wound healing; C2, IFN-γ dominant; C3, inflammatory; C4, lymphocyte depleted; C5, immunologically quiet; C6, TGF-β dominant. (*p* < 0.05 was considered significant, *p < 0.05, **p < 0.01, and ***p < 0.001).

The relationship between SERPINE family genes expression and specific immune cells infiltration in human cancers, such as B cells, dendritic cells (DCs), CD4^+^ and CD8^+^ T cells, mast cells, neutrophils, natural killer (NK) cells, and macrophages, were identified. Based on R (correlation coefficient) > 0.5 and *p* < 0.05, the results are shown in Figure 7. The results shown that SERPINE1 expression is negatively related to B cells in TGCT. SERPINE2 expression was significantly positive association with Macrophages in TGCT and negative related to Monocytes in UVM. Besides, SERPINE2 had a positive correlation with B cells in PAAD (Figure 7A).

TMB is an emerging clinical biomarker that may predict response to immune checkpoint inhibitors (ICIs) (Choucair et al., 2020). Therefore, we further identified the correlation between SERPINE family gene expression and TMB. SERPINE1 expression was related to the TMB in seven cancers. Positive correlation was found between SERPINE1 and TMB in UCEC, THYM, SARC, and LGG, whereas negative correlation was found in STAD, LUSC, and BRCA. Besides, SERPINE2 expression positively linked to the TMB in four cancers, including TGCT, KIRP, KIRC, and KICH, while it negatively related to the TMB in THCA, STAD, PAAD, LUSC, LIHC, LGG, HNSC, ESCA, COAD, and BLCA. Positive relation between SERPINE3 and TMB was in THYM, READ, and CHOL, however, negative correlation between SERPINE3 and TMB was in THCA (Figure 7B).

### 3.7 Correlation between SERPINE family genes expression and immune subtype

Aiming to analyze the relation between SERPINE family genes expression and immune subtype, we conducted the correlation analysis. The result indicated that the expression of all SERPINE gene family members were significantly correlated with immune subtype C1 (wound healing), C2 (IFN-γ dominant), C3 (inflammatory), C4 (lymphocyte depleted), C5 (immunologically quiet), and C6 (TGF-β dominant) in pan-cancer (Thorsson et al., 2018; Goldman et al., 2020) (Figure 7C). Next, we chose four types of cancer (KICH, HNSC, COAD, BRCA) from TCGA to analyze the correlation between the expression of SERPINE family genes and immune subtype (Supplementary Figure S2). The expression of SERPINE1 and SERPINE2 were significantly associated with immune subtype in KICH. SERPINE1 was higher expressed in C1. Similarly, the expression of SERPINE2 upregulated in C1. Furthermore, we found that SERPINE1 and SERPINE2 were related to the immune subtype in COAD. SERPINE1 and SERPINE2 were higher expressed in C6. In addition, SERPINE1, SERPINE2, and SERPINE3 were all associated with immune subtype in BRCA. The expression of SERPINE1 and SERPINE2 were higher in C6. Meanwhile, SERPINE3 was higher expressed in C3.

### 3.8 Functional and pathway analyses of SERPINE family genes in human cancers

**FIGURE 8:**
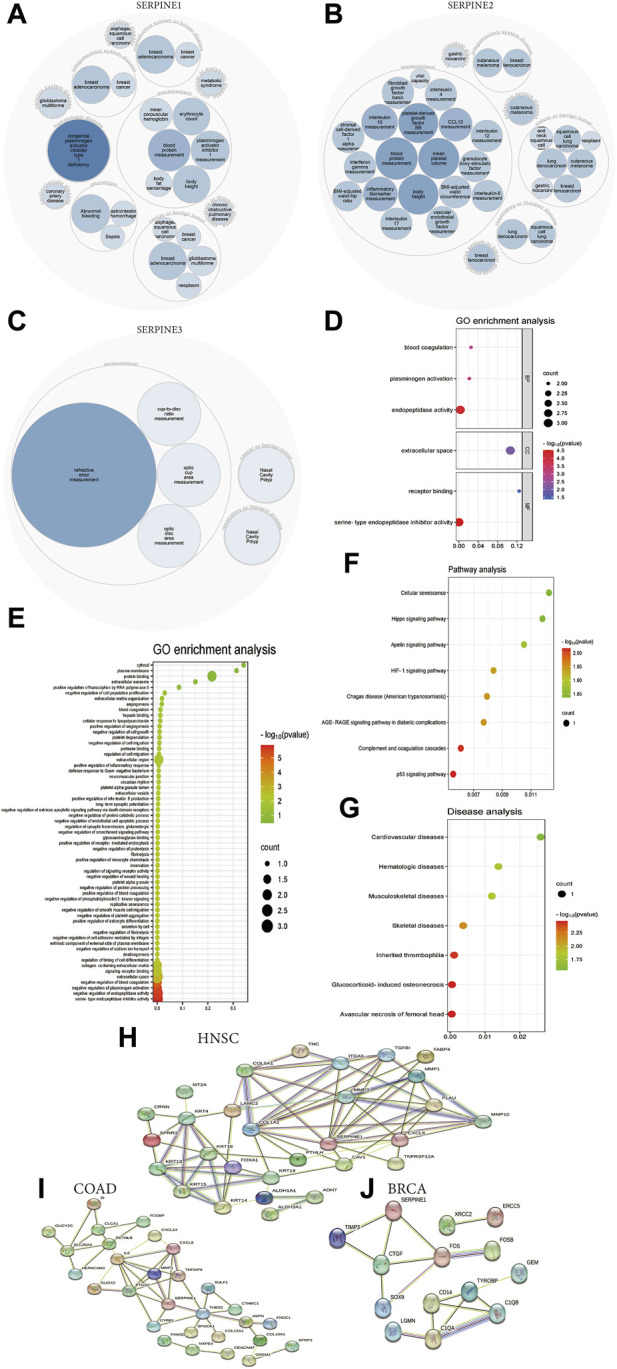
Functional and pathway annotation of SERPINE family genes in pan-cancer. **(A)** SERPINE1 related diseases network. **(B)** SERPINE2 related diseases network. **(C)** SERPINE3 related diseases network. **(D)** GO enrichment analysis from DAVID database. **(E)** GO enrichment analysis from KOBAS database. **(F)** Pathway analysis from KOBAS database. **(G)** Diseases analysis from KOBAS database. **(H)** SERPINE1-binding proteins in HNSC from STRING database. **(I)** SERPINE1-binding proteins in COAD from STRING database. **(J)** SERPINE1-binding proteins in BRCA from STRING database.

We could find SERPINE family gene-related diseases network from OPENTARGET platform. SERPINE1 is highly associated with hematologic disease, integumentary system disease, nervous system disease, cardiovascular disease, reproductive system or breast disease, gastrointestinal disease, and respiratory or thoracic disease (Figure 8A). Besides, SERPINE2 is associated with respiratory or thoracic disease, integumentary system disease, endocrine system disease, and reproductive system or breast disease (Figure 8B). SERPINE3 is related to respiratory or thoracic disease (Figure 8C).

Next, we performed GO functions and KEGG pathways associated with SERPINE genes in human cancers. The enrichment analysis of KEGG showed that the top three enriched pathways associated with SERPINE genes expression included P53 signaling pathway, complement and coagulation cascades, and AGE-RAGE signaling pathway in diabetic complications (Figure 8F). Besides, typical enriched GO items, including biological process (BP), cellular components (CC), and molecular functions (MF) were shown in Figure 8D, which were based on DAVID database. Moreover, GO enrichment analysis indicated all cell biological functions from KOBAS database. SERPINE genes significantly related to serine-type endopeptidase inhibitor activity (Figure 8E). KEGG disease analysis found that the top three diseases related to the SERPINE genes expression included avascular necrosis of femoral head, glucocorticoid-induced osteonecrosis, and inherited thrombophilia (Figure 8G).

In order to research the molecular mechanism of SERPINE1 in tumorigenesis, we applied the STRING database to investigate SERPINE1-binding proteins. There are 12 SERPINE1-binding proteins in HNSC, including COL1A1, COL5A1, ITGA5, MMP3, TGFBI, FABP4, MMP1, PLAU, MMP10, CXCL8, TNFRSF12A, and CAV1 (Figure 8H). We also obtained 6 SERPINE1-binding proteins in COAD, including PTGS2, IL6, MMP1, CXCL8, THBS2, and CYR61 (Figure 8I). Besides, 3 SERPINE1-binding proteins were found in BRCA, including TIMP3, CTGF, and FOS (Figure 8J).

### 3.9 Drug sensitivity analysis

**FIGURE 9:**
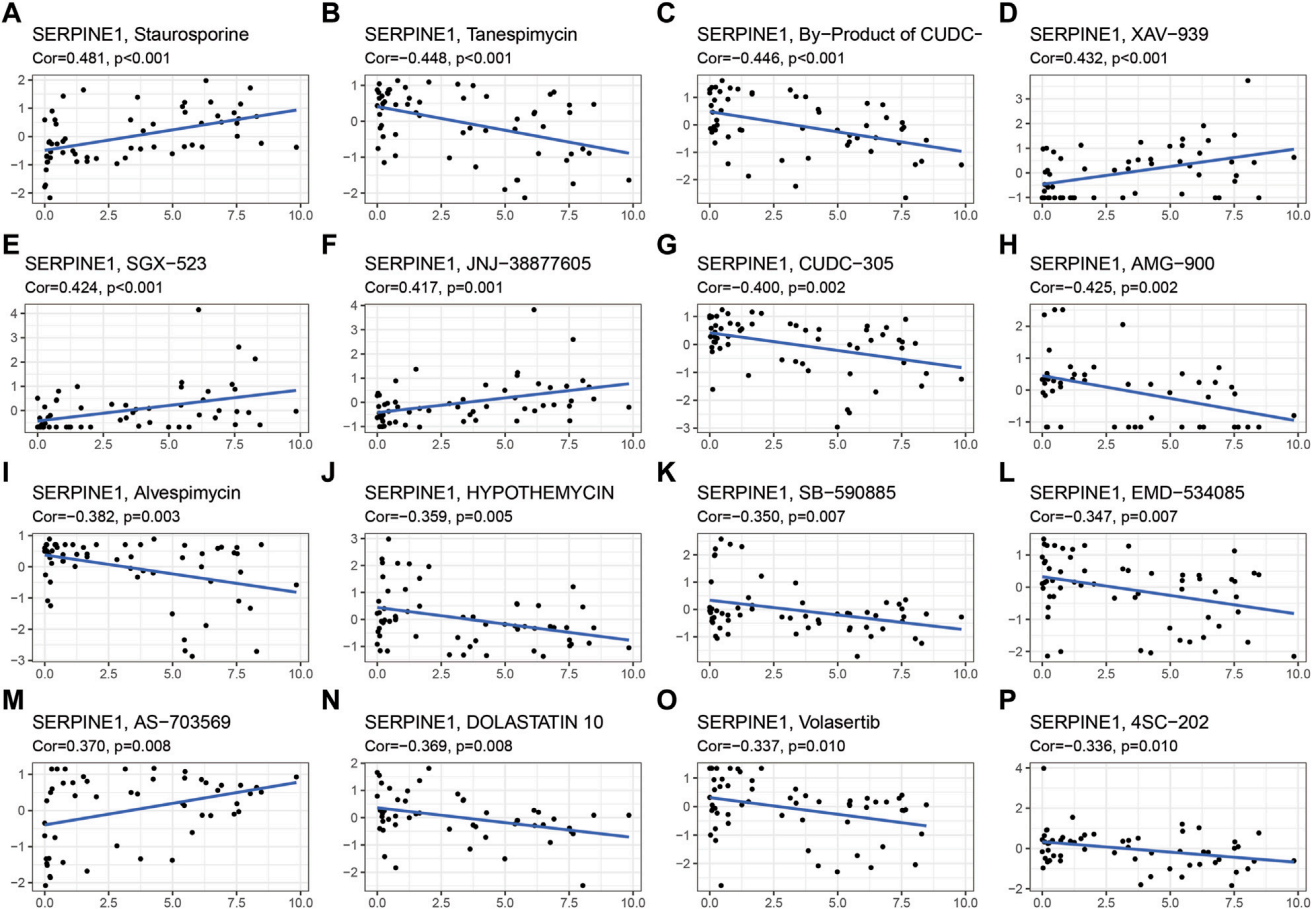
Drug sensitivity analysis of SERPINE family genes. Cor, correlation coefficient.

Aiming to explore the potential correlation between drug sensitivity and SERPINE family genes expression in various human cancer cell lines from the CellMiner™ database, the integrated analysis was performed (Figure 9). The result indicated that SERPINE1 expression had a positive relationship with drug sensitivity of Staurosporine, XAV-939 (an inhibitor of the Wnt/β-catenin pathway) (Huang et al., 2021), SGX-523 (MET-specific small-molecule kinase inhibitor) (Zhang et al., 2010), JNJ-38877605 (MET kinase inhibitors) (Pennacchietti et al., 2014). In addition, SERPINE1 expression was negatively associated with the drug sensitivity of Tanespimycin (also called 17-AAG, an HSP90 inhibitor) (Gaspar et al., 2009), By-Product of CUDC-305 (an HSP90 inhibitor), CUDC-305 (also called Debio 0932, an HSP90 inhibitor) (Isambert et al., 2015), AMG-900 (a pan Aurora Kinase inhibitor) (Fischer et al., 2021), Alvespimycin (also called 17-DMAG, an HSP90 inhibitor) (Li L. et al., 2020), HYPOTHEMYCIN, SB-590885 (a B-Raf kinase inhibitor) (King et al., 2006), EMD-534085 (the, E.g.,5 inhibitor) (Hollebecque et al., 2013), AS-703569 (also called Cenisertib), DOLASTATIN 10 (a broad-spectrum antitubulin anticancer pentapeptide) (Singh, 2022), Volasertib (also called BI 6727, a PLK1 inhibitor) (Zhang et al., 2022b), and 4SC-202 (also called Domatinostat, a HDAC inhibitor) (Bretz et al., 2019). The results may have implications for clinical medication based on the drug sensitivity of SERPINE family genes.

## 4 Discussion

In view of the important roles of SERPINE gene family in progression of a variety of tumors, it is vital to research the expression patterns and prognostic values and immunological roles in human cancers, which could contribute to cancer diagnosis and treatment. In the present study, we firstly performed basal expression levels of SERPINE family genes in 33 cancer types, and pericancerous or normal tissues from TCGA. We found SERPINE1 was significantly upregulated in nine cancer types compared with normal tissues, while SERPINE1 expression was downregulated in four cancer types. Whereas the abnormal expression of SERPINE2 was related to fifteen different cancers, twelve of which were upregulated and three were downregulated. SERPINE3 is higher expressed in six cancer types and low expressed in seven cancers. Furthermore, abnormal expression of SERPINEs was observed in multiple tumors tissues compared to normal tissues in TCGA and GTEx databases. Our last differential comparison was based on the SERPINEs protein levels analysis in normal and tumor tissues. SERPINE1 levels were established to be elevated in HNSC, KIRC, LIHC, PAAD, as well as GBM and suppressed in BRCA. The trend of elevated SERPINE2 expression in tumor tissues was observed in COAD and PAAD. We also summarized all meaningful survival-related results from different databases. The SERPINEs were significantly related to patient survival of some cancers. Besides, SERPINE1 was a detrimental factor in HNSC, STAD, LUSC, CESC, SARC, PAAD, KIRP, LUAD, which was consistent in different databases and was consistent with most previous studies (Guo et al., 2022; Hong et al., 2022; Minemura et al., 2022; Xu et al., 2022; Zhou et al., 2022; Wang et al., 2023; Zhai et al., 2023). We also found that the overexpression of SERPINE2 was significantly associated with worse OS in MESO, BLCA, and LIHC. Previous studies demonstrated that high SERPINE2 expression was related to worse OS, adverse pathologic features and may serve as a prognostic biomarker for bladder cancer (Li F. et al., 2020; Chuang et al., 2021), which was consistent with our findings. Upregulation of SERPINE3 expression was associated with worse prognosis in KIRC, LIHC, and UCEC. In general, these results strongly indicate that SERPINE family genes can serve as great prognosis-related markers in pan-cancer.

Recent studies demonstrated that TME may have dramatic effects on tumor proliferation, metastasis, micro angiogenesis, and immune escape (Altorki et al., 2019; Sautès-Fridman et al., 2019; Fane and Weeraratna, 2020). Our results indicated that SERPINE family gene expression was correlated to the TME in pan-cancer. Specially, SERPINE1 and SERPINE2 expression had a positive correlation with the levels of stromal score and immune score, suggesting SERPINE1 and SERPINE2 are predicted to efficient immunomodulatory factors. In addition, the results of transcriptome analysis in pan-cancer indicated that the immune components in TME contribute to the prognosis of patients. Besides, the stromal and immune components in TME are significantly associated with KICH, HNSC, COAD, and BRCA. Chen et al. demonstrated that TME played a pivotal role in RCC progression or metastasis and affected the response to immune or targeted therapy in clinical cohorts. More importantly, SERPINE2 was identified as a gene participating in the metastasis process, and found that SERPINE2 was highly-expressed in RCC as a differential expressed gene which could potentially predict metastasis, suggesting that it may prove to be a novel target to advanced or metastatic RCC (Chen et al., 2023). Zhou et al. indicated that SERPINE1 is likely to be involved in TME remodeling and may also contribute to the immune evasion process in the occurrence and development of HNSC, which affect the response to immune therapy for HNSC (Zhou et al., 2022). Wang et al. applied five genes, including SERPINE1, to construct a tumor microenvironment immune gene (TMEIG) score system, which can accurately predict the prognosis and immune checkpoint blockade response of colorectal cancer patients. Meanwhile, SERPINE1 may be a potential target related to immunotherapy (Wang et al., 2022). Smirnova et al. suggested that SERPINE2 is required in the extracellular milieu of tumors where it acts in multiple ways to regulate tumor matrix deposition, thereby controlling tumor cell dissemination in breast cancer (Smirnova et al., 2016). These results indicate the potential value of SERPINE family genes as therapeutic targets in some cancers, but multiple correlation studies are needed to prove its feasibility. Therefore, it is important to explore the interaction between tumor cells and immune cells in order to provide new and effective treatment options for patients. We also found the positive or negative correlation between SERPINE family gene and Stemness score in pan-cancer, especially in HNSC. We think that this correlation analysis between SERPINEs and DNAss and RNAss may be useful for patients who have a response to stem cell-based immunotherapy. Furthermore, the relationship between SERPINE gene expression and tumor stages was analyzed in selected cancers from different databases, where the results indicated that *SERPINE1* and *SERPINE2* expression were significantly increased in advanced-stage COAD. These results also encouraged us to think the roles of SERPINEs as diagnosis and prognosis markers in COAD.

The immune infiltration analysis was conducted. Our results indicated that SERPINEs expression was significantly associated with immune infiltration levels, such as B cells, Macrophages, and Monocytes. In particular, SERPINE2 showed a significantly correlation with immune cells in TGCT and UVM. These results implicated that SERPINEs may be participated in tumor immunity regulation by mediating immune infiltration. Zhou et al. results also shown that SERPINE1 expression levels in HNSC was positively correlated with the immune invasion levels of CD4 T cells, macrophages, neutrophils, and dendritic cells, but negatively correlated with the infiltration of B cells and CD8 T cells. Thus, attenuating SERPINE1 expression can potentially enhance the infiltration of CD8 T cells and B cells and decrease the infiltration of CD4 T cells and macrophages, which would be a promising direction for improving patient outcomes and developing targeted therapeutics for HNSC (Zhou et al., 2022). TMB is an emerging independent predictor of treatment response to ICIs for immunotherapy in pan-cancer and high TMB is correlated with longer survival after treatment with ICIs (Boumber, 2018; Boichard et al., 2020). Due to the significant correlation between SERPINEs and TMB, we suggest that SERPINEs might have predictive value in immunotherapy. Furthermore, the potential association between SERPINE family gene expression and immune subtypes was also analyzed in pan-cancer and selected cancer types (KICH, HNSC, COAD, and BRCA). Our results showed that all SERPINEs were significantly related to immune subtypes in pan-cancer, and most of SERPINEs had a close correlation with KICH, COAD, and BRCA.

The roles of SERPINEs in cancer progression were explored via enrichment analysis. The analysis revealed that SERPINEs were related to many diseases, such as hematologic disease, cardiovascular disease, and respiratory or thoracic disease. Besides, the results showed that SERPINE genes significantly related to serine-type endopeptidase inhibitor activity and endopeptidase activity, indicating that SERPINEs might be related to tumor progression. KEGG analysis obtained herein demonstrated that SERPINEs may influence the tumorigenesis, progression, metastasis, and drug resistance of cancer by taking part in the processes of P53 signaling pathway (Lei et al., 2022; Asl et al., 2023; Fang et al., 2023) and HIF-1 signaling pathway (Fang et al., 2023). In brief, these pathways may be helpful for the research of the relation between SERPINEs and targeted drug in the future.

SERPINE family genes were abnormally expressed in different cancer types and were closely associated with tumor immunity. Based on our analysis, we found that SERPINE family genes can be used as prognostic biomarkers for diverse tumors, including the prognostic value of SERPINE1 in HNSC, STAD, LUSC, CESC, SARC, PAAD, KIRP, and LUAD, SERPINE2 in MESO, BLCA, and LIHC, and SERPINE3 in KIRC, LIHC, and UCEC. However, to date, there are few studies on the survival analysis of SERPINE family genes in these tumors. In the future, the prognostic value of SERPINE genes and the exploration of SERPINE genes as prognostic biomarkers in these tumors should be strengthened. In addition, our results suggest that SERPINE1 and SERPINE2 can serve as diagnostic biomarkers for COAD. Most importantly, due to the significant correlation between SERPINEs and TME, TMB, and immune cell infiltration, combined with previous research results, we believe that SERPINEs have potential value in predicting immunotherapy response and may be a novel immunotherapy target for diverse cancers, especially for KICH, HNSC, COAD, and BRCA. Therefore, our results provide a new direction for the follow-up clinical research and tumor immunotherapy.

Hypoxia is a crucial characteristic of tumor microenvironment, and HIF-1 is a key transcription factor that is induced by hypoxia, which regulates the expression of genes that lead to angiogenesis, metabolic reprogramming, extracellular matrix remodeling, epithelial-mesenchymal transition, motility, invasion, metastasis, cancer stem cell maintenance, immune evasion, and resistance to chemotherapy and radiation therapy (Carmeliet et al., 1998; Hanahan and Weinberg, 2000; Harris, 2002; Schito and Semenza, 2016). In hypoxic environment, HIF-1 can regulate SERPINE1 gene transcription (Jung et al., 2011; Peterle et al., 2018; Zhang et al., 2023). Our results found that SERPINE1 can be used as prognostic biomarkers in multiple cancer types, including HNSC, STAD, LUSC, CESC, SARC, PAAD, KIRP, and LUAD. Furthermore, by TME, TMB, and immune cell infiltration correlation analysis, we discovered that SERPINE1 can be used as a new therapeutic target for a variety of tumors. Therefore, our results indirectly reflect the link between hypoxia and tumor. The current study may provide new insights into how hypoxia affects cancer prognosis and may help guide targeted therapies for specific cancer types.

However, the limitation was still in existence based on these bioinformatic analyses. First, some contradictory results of individual cancers were uncovered. Second, the sample size was small and we needed to test and validate the expression and function of SERPINEs in a larger sample cohort. Third, further *in vitro* or *in vivo* biological experiments were necessary to verify these conclusions and enhance clinical application.

## 5 Conclusion

In conclusion, the present research revealed complex and comprehensive roles of SERPINE family gene members expression in tumor progression and clinical outcomes, demonstrating that SERPINE family genes could serve as promising prognostic biomarkers in special cancer types. The SERPINE family genes were also related to TME and stemness score, infiltration of immune cells, TMB, and immune subtypes, which providing a few directions for immunotherapy and new insights for further research of the SERPINE family genes as potential targets in tumors. This study also deepens the understanding of the relationship between hypoxia and tumors, and provides new evidence for SERPINE1 as a therapeutic target for a variety of tumors.

## Data Availability

The original contributions presented in the study are included in the article/Supplementary Material, further inquiries can be directed to the corresponding author.
