# Supplementary material for: Pan-cancer analysis of SERPINE family genes as biomarkers of cancer prognosis and response to therapy
Source: Front Mol Biosci. 2024 Jan 11;10:1277508. doi: 10.3389/fmolb.2023.1277508 (PMC10808646; doi:10.3389/fmolb.2023.1277508)
Supplement: Supplementary file 1 [file DataSheet2.PDF]

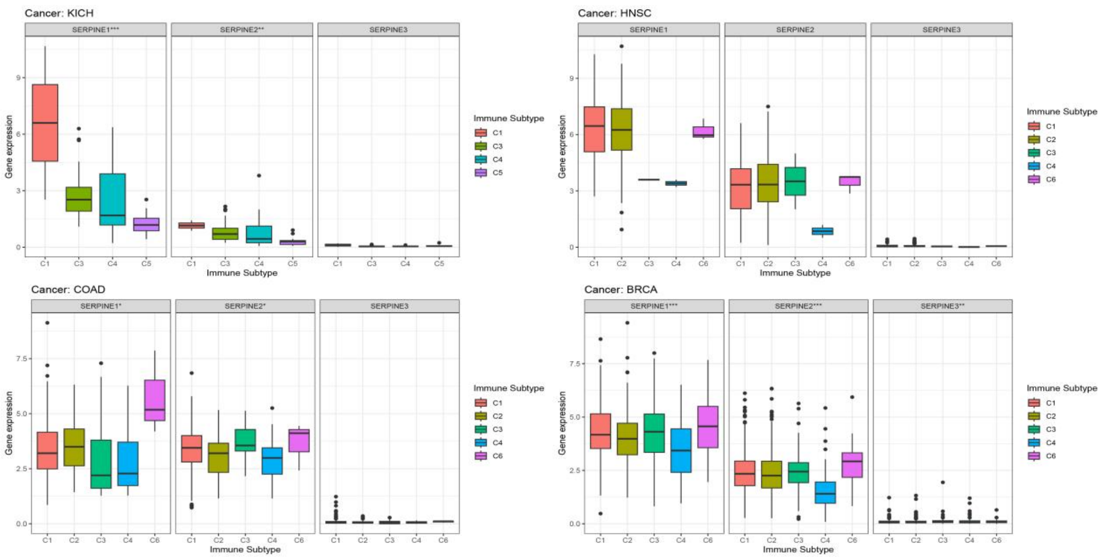

Figure S2.

SERPINE family genes expression levels of different immune subtype in four cancer types.
