# Supplementary material for: Pan-cancer analysis of SERPINE family genes as biomarkers of cancer prognosis and response to therapy
Source: Front Mol Biosci. 2024 Jan 11;10:1277508. doi: 10.3389/fmolb.2023.1277508 (PMC10808646; doi:10.3389/fmolb.2023.1277508)
Supplement: Supplementary file 2 [file DataSheet1.PDF]

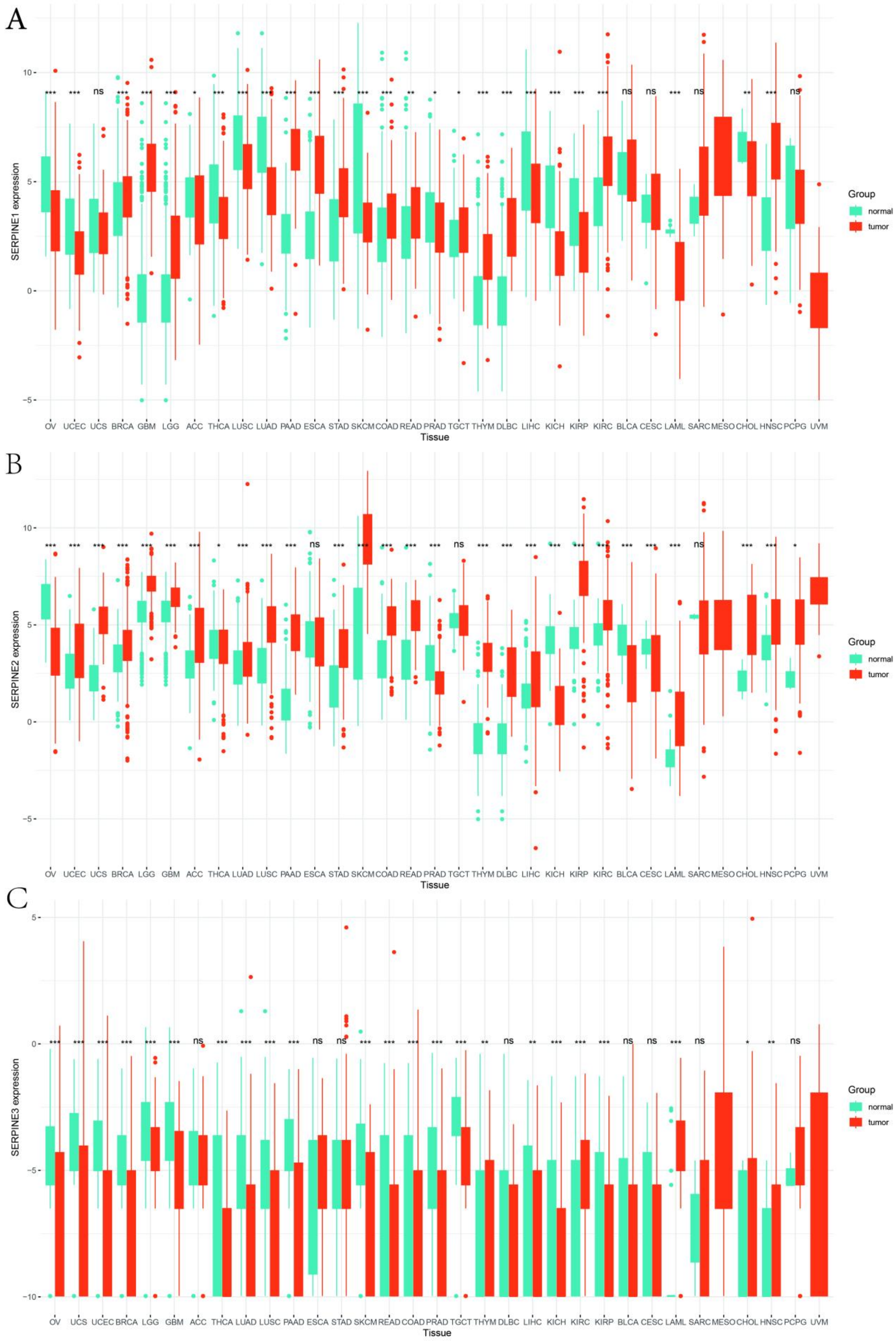

Figure S1.

Differential expression of SERPINEs in human pan-cancer according to The Cancer Genome Atlas (TCGA) and Genotype-Tissue Expression (GTEx) databases. *p < 0.05, **p < 0.01, ***p < 0.001.
